# EFFECTS OF PHYSICAL EXERCISE DURING HOSPITALIZATION IN CHILDREN AND ADOLESCENTS WITH CANCER: A SYSTEMATIC REVIEW

**DOI:** 10.1590/1984-0462/2021/39/2019313

**Published:** 2020-10-05

**Authors:** Scárlat da Silva Santos, Luciane Dalcanale Moussalle, João Paulo Heinzmann-Filho

**Affiliations:** a Centro Universitário Cenecista de Osório, Osório, RS, Brazil.; b Universidade Federal de Ciências da Saúde de Porto Alegre , Porto Alegre, RS, Brazil.

**Keywords:** Cancer, Exercise, Hospitalization, Neoplasms, Pediatrics, Câncer, Exercício, Hospitalização, Neoplasia, Pediatria

## Abstract

**Objective:**

To identify the effects of exercise programs during hospitalization on children and adolescents with cancer.

**Data source:**

This is a systematic review, carried out in PubMed/ Medical Literature Analysis and Retrieval System Online (MEDLINE), Latin American and Caribbean Health Sciences Literature (LILACS), Scientific Electronic Library Online (SciELO), Latin American and Caribbean Center on Health Sciences Information (BIREME), and Physiotherapy Evidence Database (PEDro). We selected studies that included children and adolescents diagnosed with cancer (solid or hematologic) and submitted to exercise protocols during hospitalization. Studies involving patients with other pathologies or with a medical contraindication for exercise were excluded. We used the following search strategy: Neoplasm OR Leukemia OR Cancer OR Tumor OR Medical Oncology AND Hospitalization OR Inpatient Care Units OR Intrahospital AND Exercise. The methodological quality of the studies was analyzed by the PEDro scale.

**Data synthesis:**

Among the 626 articles found, only 9 fulfilled the inclusion criteria, obtaining a regular methodological quality. The samples had 172 participants, aged 4 to 18 years. Only 6 studies presented both intervention group and control group. The intervention group received strength, aerobic, and muscle stretching exercises, and games, among others. The control group received the standard treatment. The studies varied regarding time, frequency, intensity, and type of exercise. Most studies showed an increase in muscle strength (4/5), followed by an improvement in physical fitness (2/3) and functional capacity (2/4). No adverse events were reported during the interventions. The methodological quality was considered regular.

**Conclusions:**

The findings suggest that. during hospitalization of children and adolescents with cancer, exercise improves muscle strength, physical fitness, and functionality.

## RESUMO

**Objetivo:**

Identificar os efeitos de programas de exercício físico durante a hospitalização de crianças e adolescentes com câncer.

**Fontes de dados:**

Trata-se de uma revisão sistemática, realizada nas bases de dados PubMed/Sistema Online de Busca e Análise de Literatura Médica (MEDLINE), Literatura Latino-Americana e do Caribe em Ciências da Saúde (Lilacs), Biblioteca Eletrônica Científica Online (SciELO), Biblioteca Regional de Medicina (BIREME) e Physiotherapy Evidence Database (PEDro). Foram selecionados estudos que incluíram crianças e adolescentes com câncer (sólido ou hematológico) que se submeteram a protocolos de exercício físico durante a hospitalização. Em contrapartida, foram excluídas pesquisas que envolveram pacientes com outras patologias ou com contraindicação médica para o exercício físico. Utilizou-se a seguinte estratégia de busca: *Neoplasm OR Leukemia OR Cancer OR Tumor OR Medical Oncology AND Hospitalization OR Inpatient Care Units OR Intrahospital AND Exercise*. A qualidade metodológica foi analisada pela escala PEDro.

**Síntese dos dados:**

De um total de 626 artigos, somente nove foram incluídos. As amostras totalizaram 172 participantes, com idade entre 4 e 18 anos. Apenas seis artigos apresentaram tanto grupo intervenção como grupo controle. A intervenção consistiu em exercícios de força, aeróbicos, de alongamento muscular, de jogos, entre outros. Já o controle realizou o tratamento padrão da doença. Os protocolos variaram quanto ao tempo, à frequência, à intensidade e ao tipo de exercício. A maioria dos estudos evidenciou aumento na força muscular (4/5), seguido de benefícios na aptidão física (2/3) e na funcionalidade (2/4). Não foi relatado nenhum evento adverso durante as intervenções. A qualidade metodológica foi considerada regular.

**Conclusões:**

Os achados sugerem que o exercício físico melhora a força muscular, a aptidão física e a funcionalidade de crianças e adolescentes com câncer durante a hospitalização.

## INTRODUCTION

Cancer is a degenerative disease, resulting from the accumulation of lesions in the genetic material of cells, which can affect any part of the organism.^1^ The disease is characterized by its severity, putting the life of the individual at risk, with no age or gender predisposition. Clinically, it causes problems such as pain, weight loss, reduced energy, nodule growth, among others.^2^

Estimates from the International Agency for Research on Cancer (IARC) show that the global incidence of childhood cancer has increased in recent decades. Worldwide, about 215,000 types of cancer are diagnosed annually in children under 15 years of age and approximately 85,000 in individuals aged 15 to 19 years. These projections are based on data collected from over 100 population records of cancer incidence in 68 countries, from 2001 to 2010.^3^ In Brazil, estimates predicted 12,500 new cases of cancer in children and adolescents for each year of the 2018-2019 biennium, 1,300 of them in the South Region.^4^ ^,^ ^5^

Childhood cancer has a different clinical and histological presentation from that of adults, and its causes are not yet well-defined. Signs and symptoms include typical nodules, pallor, generalized weakness, progressive pain, fever, impaired vision, and loss of appetite. The most common types of cancer in this age group are leukemias, which present the highest incidence (26%), followed by lymphomas (14%), and tumors of the central nervous system (13%).^3^ ^,^ ^4^ Acute lymphoblastic leukemia (ALL) is the most common childhood malignancy, representing about 30% of cancer cases in children under 15 years of age.^3^ ^,^ ^6^ Mortality rate depends on the development of the disease, the child’s age, and the initial response to treatment.^4^ ^,^ ^5^

Advances in treatment techniques, such as the combination of chemotherapy and radiotherapy, contribute to the high survival rate (~ 70%) of children during therapy;^4^ ^,^ ^7^ however, treatment- and cancer-related adverse effects have increased in the short-, medium-, and long-term. In the short-term, side effects are very similar to those of adults, with the onset of nausea, vomiting, and predisposition to other infections. In the medium- and long-term, they influence motor development, weight-for-height growth, musculoskeletal/functional performance, and quality of life (QOL).^5^ ^,^ ^8^

The treatment of the disease requires repeated and prolonged hospitalizations, which involve various types of related stressors, especially invasive procedures and bed restriction.^9^ These limitations, caused by the disease and the treatment, directly affect the patients’ physical activity levels, leading to excessive rest and consequent physical deconditioning.^8^ ^,^ ^10^ ^,^ ^11^ In contrast, directed exercise performed for a few weeks during treatment is associated with an improvement in psychological, physiological, and physical aspects, highlighting the potential positive effect of this type of intervention.^7^ ^,^ ^9^ ^,^ ^12^ ^,^ ^13^ Some studies show that exercises performed at the hospital/home result in benefits to muscle strength, physical fitness, and functional capacity.^14^ ^,^ ^15^ ^,^ ^16^ ^,^ ^17^ ^,^ ^18^ ^,^ ^19^ ^,^ ^20^ ^,^ ^21^

To date, we have found no critical or systematic reviews with the purpose of exclusively investigating the effects of exercise during hospitalization on children and adolescents with cancer, which justifies this study. Identifying the real effects of exercise and reflecting on similar protocol characteristics can help professionals involved in the care of these children, in addition to increasing adherence to this type of intervention in hospitalization services. Thus, this study aimed to identify the effects of exercise programs during hospitalization on children and adolescents with cancer.

## METHOD

This systematic review was carried out according to recommendations from the Preferred Reporting Items for Systematic Reviews and Meta-Analyses.^22^

### Data sources

This is a systematic review performed in the following databases: PubMed via the Medical Literature Analysis and Retrieval System Online (MEDLINE), Latin American and Caribbean Health Sciences Literature (LILACS), Scientific Electronic Library Online (SciELO), Latin American and Caribbean Center on Health Sciences Information (BIREME), and Physiotherapy Evidence Database (PEDro).

### Study selection

We selected intervention studies (clinical trial and/or quasi-experimental research) in English, Portuguese, and Spanish, with no filter as to age or year of publication of the articles. The study selection took place in October 2019, based on nine keywords combined with Boolean operators. In addition, potential articles were manually selected to compose the present work by searching the references of each research.

### Search strategy

The following search strategy was used: “Neoplasm OR Leukemia OR Cancer OR Tumor OR Medical Oncology AND Hospitalization OR Inpatient Care Units OR Intrahospital AND Exercise.” All terms are controlled keywords registered in the Health Sciences Descriptors (DeCS), with the exception of the word “Intrahospital.” We decided to keep this word because many studies use this term in abstracts. All terms searched should be part of at least the title, abstract, or keywords.

### Inclusion and exclusion criteria

Studies with children and adolescents (aged 4 to 18 years) with some type of cancer (solid or hematologic) and submitted to exercise protocols (according to each author) during hospitalization were included. On the other hand, abstracts, dissertations, theses, guidelines, editorial letters, review articles, case reports, expert opinions, and research that included patients with other pathologies (asthma, cystic fibrosis, among others) were excluded. We also excluded studies with subjects after organ transplantation, post-radiotherapy/chemotherapy sessions, or who had medical contraindication for exercise.

### Data extraction

After identifying the descriptors in the title, abstract, and/or keywords, we read the abstracts of the selected articles to assess the adequacy regarding the eligibility criteria (detailed in the previous item). The full texts of studies that fulfilled the predetermined criteria were acquired for analysis and data extraction. Two evaluators independently conducted the search and analysis of the article, and a third researcher solved any disagreements by consensus.

The following study characteristics were recorded: name of the first author, year of publication, country of data collection, age group, sample size, type of cancer, risk of mortality, treatment onset, groups studied, proposed evaluations, evaluated outcomes (muscle strength, quality of life, functional capacity, physical fitness, body composition, physical activity level, balance, fatigue, range of motion, and cardiac function), characteristics of exercise protocols, frequency, duration, and the main results found.

### Methodological quality assessment

The methodological quality was analyzed by two evaluators, and any disagreement was resolved by consensus. We used the PEDro scale based on the Delphi method, which aims to assist users regarding the methodological quality of intervention studies (criteria 2 to 11). This qualification corresponds to the number of criteria met. Item 1 was not considered in the score, as it assessed the criterion regarding the eligibility of participants, referring to the external validity of the study. Therefore, scores ranged from 0 to 10.^23^

## RESULTS

**Figure 1:**
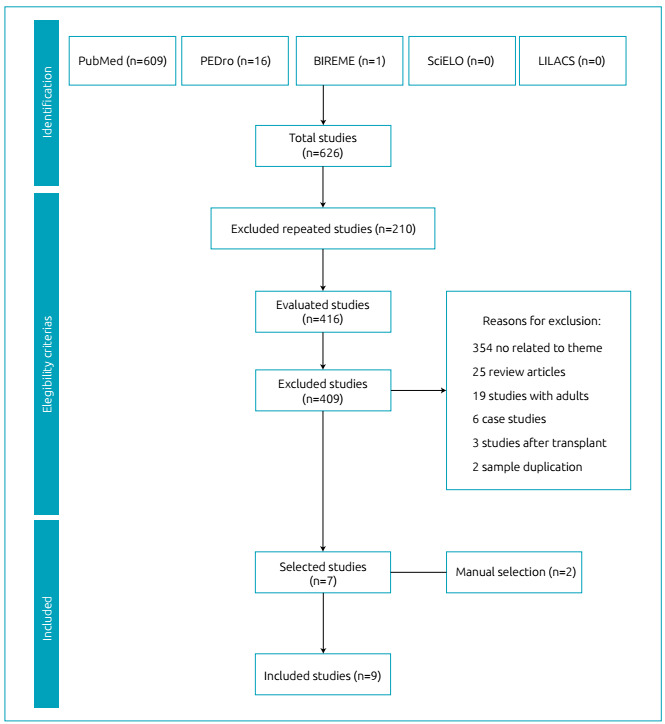
Systematization of the studies selected in the review.

Out of a total of 626 articles, 609 were found in PubMed, 16 in PEDro, one in BIREME, and none in SciELO and LILACS. Among these articles, 210 were excluded because they were duplicated in the databases, with 416 documents remaining. Subsequently, 409 studies were discarded, as they did not meet the eligibility criteria, with only seven articles remaining. In addition, two other articles were selected through manual search of study references. Thus, this review comprised nine articles (Figure 1).

Seven (77.7%) out of the nine studies were conducted in Europe, one in South America (11.1%), and one in Oceania (11.1%). In total, 172 participants were selected, with the sample size ranging from seven to 68 subjects. The age of the individuals ranged from 4 to 18 years. The patients included had different types of cancer, most of them hematologic, with predominance of ALL. Treatment onset varied between six and 24 months (Table 1).

**Table 1:** Identification and characteristics of the included studies.

Authors	Country	Age group (years)	Sample	Type of cancer	Mortality risk	Treatment onset (months)
Morales et al.^18^	Spain	4-18	68	Solid tumors and leukemia	-	-
Fiuza-Luces et al.^20^	Spain	4-18	9	Extracranial solid tumor	-	-
Fiuza-Luces et al.^21^	Spain	4-16	24	Extracranial solid tumor	-	-
Bogg et al.^17^	Australia	6-17	14	ALL, AA, ALCL, AML, MPD	-	-
Perondi et al.^6^	Brazil	5-16	6	ALL	Low or High	> 6
Speyer et al.^9^	France	9-18	30	Hematological malignancy, solid tumors, undetermined	-	-
Ruiz et al.^15^	Spain	5-16	7	ALL, AML, rhabdomyosarcoma, neuroblastoma	High	-
Chamorro-Viña et al.^16^	Spain	4-7	7	ALL	Intermediate	18-24
San Juan et al.^7^	Spain	4-7	7	ALL	Intermediate	18-24

ALL: acute lymphoblastic leukemia; AA: aplastic anemia; ALCL: acute large cell lymphoma; AML: acute myeloid leukemia; MPD: myeloproliferative disorder; -: not informed.

Regarding the evaluations performed, five (55.5%) studies assessed the muscle strength of upper limbs (UL) and lower limbs (LL), mostly through 5-, 6- and, or 10-repetition maximum tests. QOL was also assessed in five (55.5%) studies, of which three (3/5) administered the pediatric QOL questionnaire. Functional capacity was evaluated in four (44.4%) investigations, three (3/4) by the Timed Up and Go test (TUG) and one (1/4) by the 6-minute walk test (6MWT). Also, physical fitness was measured in three articles (33.3%) using cardiopulmonary exercise testing. Only six studies (66.6%) used a control group (Table 2).

**Table 2:** Characteristics of the studied outcomes and the tests evaluated in this review.

Authors	Evaluated groups	Proposed evaluations	Tested outcomes
Morales et al.^18^	G1 G2	Echocardiogram	Cardiac function
Fiuza-Luces et al.^20^	G1 G2	Blood collection Triaxial accelerometer	Immune system Physical activity level
Fiuza-Luces et al.^21^	G1 G2	5RM - bench press, bent-over row, and leg press Pediatric quality of life inventory (PedsQL) TUG - 3 m and TUDS CPET BMI and lean mass Triaxial accelerometer	MS from UL and LL QOL Functional capacity Physical fitness Body composition Physical activity level
Bogg et al.^17^	IG	Dynamometer Pediatric quality of life inventory (PedsQL) 6MWT - adapted Unipedal balance on a flat surface Pediatric quality of life inventory (fatigue scale) (PedsQL)	MS from UL and LL QOL Functional capacity Balance Fatigue
Perondi et al.^6^	IG	10RM - bench press, lat pulldown, leg extension, and leg press Pediatric quality of life inventory (PedsQL)	MS from UL and LL QOL
Speyer et al.^9^	IG CG	Child health questionnaire (CHQ)	QOL
Ruiz et al.^15^	IG CG	6RM - bench press, bent-over row, and leg press TUG - 3 and 10 m and TUDS CPET	MS from UL and LL Functional capacity Physical fitness
Chamorro-Viña et al.^16^	IG CG	Weight, BMI, body fat, and lean mass	Body composition
San Juan et al.^7^	IG	6RM - bench press, bent-over row, and leg press Child health and illness profile (CHIP-CE / CRF) TUG - 3 and 10 m and TUDS CPET Goniometry	MS from UL and LL QOL Functional capacity Physical fitness Ankle ROM

IG: intervention group; CG: control group; 5RM: 5-repetition maximum test; 10RM: 10-repetition maximum test; 6RM: 6-repetition maximum test; MS: muscle strength; 6MWT: 6-minute walk test; QOL: quality of life; MR: maximum repetitions; UL: upper limbs; LL: lower limbs; BMI: body mass index; CPET: cardiopulmonary exercise testing; ROM: range of motion; PedsQL: Pediatric Quality Of Life Inventory; CHQ: Child Health Questionnaire; TUG: Timed Up and Go test; TUDS: Timed Up and Down Stairs test; CHIP-CE / CRF: Child Health and Illness Profile - Child Edition / Child Report Form.

With respect to intervention protocols, we highlight that almost all studies used a combination of strength and aerobic training, and some included balance activities, stretching, and games. The sessions lasted from 10 to 120 minutes, with frequency between two and five sessions/week. The duration of the treatment program ranged from three to 22 weeks. The vast majority of articles showed an increase in muscle strength (4/5), followed by an improvement in physical fitness (2/3) and functional capacity (2/4). Only one study (1/5) identified an improvement in QOL. Moreover, two investigations showed that muscle strength, physical fitness, functional capacity (2/2), among others, were maintained for some time (20 weeks) after the end of the study. No adverse events were reported during the interventions (Table 3).

**Table 3:** Main results of the studies included in this review.

Authors	Exercise program	Program description	Frequency, time, and duration	Main results
Morales et al.^18^	Aerobic training Strength training	Cycle ergometer, treadmill, and arm crank 1-3 sets of 6-15 repetitions - UL, LL, and trunk	2-3x/week / 60 to 70’/ 22 weeks	↑ Cardiac function
Fiuza-Luces et al.^20^	Aerobic training Strength training	Cycle ergometer, treadmill 2-3 sets of 8-15 repetitions - UL, LL, and trunk	3x/week / 60 to 70’/ 17 weeks	↔ Immune system ↔ Physical activity level
Fiuza-Luces et al.^21^	Aerobic training Strength training	Cycle ergometer, treadmill 2-3 sets of 8-15 repetitions - UL, LL, and trunk	3x/week / 60 to 70’/ 19 weeks	↑ MS of UL and LL ↔ QOL ↔ Functional capacity ↔ Physical fitness ↔ Body composition ↔ Physical activity level
Bogg et al.^17^	Aerobic training Strength training Balance exercises Stretching	Walking, cycle ergometer, interactive video game, etc. Squat, crunch, calf, bridge, etc. Unipedal balance and twister Stretching large muscle groups	5x/week / 10 to 60’/ 7 weeks	↔ MS of UL and LL ↔ QOL ↓ Functional capacity ↓ Balance ↔ Fatigue
Perondi et al.^6^	Warm-up Strength training Aerobic training Cool-down	Treadmill - 10’ 4 sets of 6-10 repetitions - UL and LL Treadmill - 20’ Stretching large muscle groups	2x/week / 60’/ 12 weeks	↑ MS of UL and LL ↑ QOL
Speyer et al.^9^	Adapted physical activities	Games, weight training, dance, interactive video game, among others	3x/week / 30’/ -	↔ QOL
Ruiz et al.^15^	Warm-up Strength training Aerobic training Cool-down	Cycle ergometer - 15’ and stretching 1 set of 8-15 repetitions - UL, LL, and trunk Cycle ergometer - 10 to 30’; running, walking, and games Cycle ergometer and stretching of large muscle groups	3x/week / 90 to 120’/ 16 weeks	↑ MS of UL and LL ↑ Functional capacity ↑ Physical fitness
Chamorro-Viña et al.^16^	Warm-up Aerobic training Strength training	UL and LL movements and stretching Cycle ergometer - 10 to 40’ 1 set of 12-15 repetitions - UL, LL, and trunk	5x/week / 50’ / ~3 weeks	↑ BMI ↑ Weight ↑ Body fat ↑ Lean mass
San Juan et al.^7^	Warm-up Strength training Aerobic training	Cycle ergometer - 15’ and stretching 1 set of 8-15 repetitions - UL, LL, and trunk Cycle ergometer - 10 to 30’; running, walking, and games	3x/week / 90 to 120’ / 16 weeks	↑ MS of UL and LL ↔ QOL ↑ Functional capacity ↑ Physical fitness ↔ Ankle ROM

MS: muscle strength; QOL: quality of life; UL: upper limbs; LL: lower limbs; BMI: body mass index; ROM: range of motion; ↑: increased; ↔: did not change; ↓: decreased; -: not informed; ~: approximately; twister: physical skill game.

The mean methodological quality score was 5.6 points, ranging from 4 to 9. The main factors that lowered the quality score were related to concealed and random allocation, blinded participants and therapists/evaluators, and comparison between groups (Table 4).

**Table 4:** Evaluation of the methodological quality of the selected studies.

Criteria evaluated	Morales et al.^18^	Fiuza-Luces et al.^20^	Fiuza-Luces et al.^21^	Bogg et al.^17^	Perondi et al.^6^	Speyer et al.^9^	Ruiz et al.^15^	Chamorro-Viña et al.^16^	San Juan et al.^7^
Eligibility criteria*	+	+	+	+	+	+	+	+	+
Random allocation	-	+	+	-	-	+	-	-	-
Concealed allocation	-	+	+	-	-	-	-	-	-
Similar groups	+	+	+	+	+	+	+	+	+
Blinded participants	-	+	+	-	-	+	-	-	-
Blinded therapists	-	-	-	-	-	-	-	-	-
Blinded evaluators	-	+	+	-	-	-	-	+	-
Adequate follow-up	+	+	+	+	+	+	+	+	+
Intention-to-treat analysis	+	+	+	+	+	+	+	+	+
Comparisons between groups	+	+	+	-	-	+	-	-	-
Point estimates and variability	+	+	+	+	+	+	+	+	+
Total score	5	9	9	4	4	7	4	5	4

*The eligibility criteria item does not contribute to the total score; +: yes; -: no.

## DISCUSSION

The present review assessed nine studies,^6^ ^,^ ^7^ ^,^ ^9^ ^,^ ^15^ ^,^ ^16^ ^,^ ^17^ ^,^ ^18^ ^,^ ^21^ ^,^ ^22^ with regular methodological quality, which investigated the effects of exercise programs during hospitalization on children and adolescents with some type of cancer. All selected articles found positive effects in the short/medium-term (three to 22 weeks), with muscle strength, physical fitness, and functional capacity having the best outcomes.

Leukemia is one of the most prevalent types of cancer in this population, especially ALL,^3^ ^,^ ^4^ which was the most frequent malignancy in selected studies that evaluated the effects of exercise.^6^ ^,^ ^7^ ^,^ ^15^ ^,^ ^16^ ^,^ ^17^ Although the cure rate has increased, there is still questioning as to the most appropriate and safe way to perform exercises.^3^ ^,^ ^6^ Despite being devised to treat various diseases, the hospital environment is not yet prepared for the performance of exercises, especially when treating children.^24^ Such obstacle may be related to age and the restricted physical space, in addition to the fact that absolute rest was recommended in the past.^6^ ^,^ ^25^ However, even with so many limitations and adversities, the results of the selected studies reinforce the need for this type of intervention. No complications were reported during the interventions, indicating that this practice can be safe in the hospital. We underline that no participant performed exercises if they had a fever, low platelet count, reduced neutrophil count, anemia, and/or medical contraindication.^7^ ^,^ ^9^ ^,^ ^15^ ^,^ ^16^ ^,^ ^17^ ^,^ ^18^ ^,^ ^21^

Muscle strength improved in most studies,^6^ ^,^ ^7^ ^,^ ^15^ ^,^ ^21^ and was assessed by the 5-repetition maximum test (5RM), 6-repetition maximum test (6RM), 10-repetition maximum test (10RM), and using a dynamometer. The main objective of including strength training in the protocols is to reduce the loss of muscle mass, commonly observed in patients with prolonged rest, as in the case of cancer.^26^ Extrapolating these findings, when we analyze the results of chronic diseases, studies show that strength training should focus on large muscle groups (biceps, triceps, and quadriceps), due to their relationship with functional capacity, performance of daily activities, and survival.^7^ ^,^ ^27^ ^,^ ^28^ In pediatric hospital practice, these muscle groups could be worked with playful activities, for example, ball games, handgrip activities, and jumping, as performed by Speyer et al., in 2010.^9^

Cardiopulmonary exercise testing is considered the gold standard for investigating physical fitness, measuring the maximal oxygen uptake.^29^ Previous findings indicate that values <32 mL/kg/min are associated with worse lung function, greater risk of hospitalization, and shorter survival in chronic diseases.^30^ ^,^ ^31^ When extrapolating our interpretation, the results of the study by San Juan et al.^7^ (24.3 mL/kg/min) are clearly below this cut-off point; however, the research by Ruiz et al.^15^ did not present the initial data for this parameter, and the study by Carmen Fiuza-Luces et al.^21^ did not report this result adjusted for body mass. San Juan et al.^7^ also identified a reduction in another marker of physical conditioning (ventilatory threshold), obtaining mean values of 15.8 mL/kg/min, that is, below normal (≥20 mL/kg/min).^32^ Although the disease causes physical impairment, most studies found that the interventions had positive effects on this outcome, which highlights the need for this type of therapy during hospitalization.

Half of the studies indicated improvement in functional capacity after interventions in these samples,^7^ ^,^ ^15^ which were evaluated by the TUG test (3 and 10 meters) and the Timed Up and Down Stairs test. Another research also assessed functional capacity with an adapted version of the 6MWT, finding no improvement in this outcome.^17^ All these investigations aimed to assess the distance covered and/or the time taken to perform certain tasks, with these indicators being considered markers of functional capacity.^7^ ^,^ ^33^ Evidence indicates that values <10 seconds for the TUG test^33^ and <577.5 meters for the 6MWT^34^ can be good cut-off points to signal greater clinical severity. In our review, the results of the TUG test were 6.3 seconds in the study by San Juan et al.^7^ and below 10 seconds in the research by Carmen Fiuza-Luces et al.,^21^ pointing to preserved functional capacity. However, the authors did not report the mean values obtained for the 6MWT.^17^ Although childhood cancer is not a chronic disease, in many situations, its treatment extends over a long period.^1^ ^,^ ^4^ In addition, due to the lack of cut-off points to classify the worst clinical severity in this sample, we decided to exceed the interpretation regarding functional capacity.

Only the study by Perondi et al.^6^ showed benefits of the exercise program to the QOL of hospitalized patients. This result diverges from that documented in adult samples,^35^ ^,^ ^36^ taking into account that both drug and physical interventions seem to improve the QOL of these patients. This finding may lead us to assume that perhaps exercise does not have a favorable impact on the perception of parents and/or individuals with the disease. Marchese et al.^13^ reported that both children and parents end up hiding the problems investigated by the questionnaires, which prevents the proper assessment of these data. Nevertheless, none of the studies reported a reduction in QOL domains,^7^ ^,^ ^9^ ^,^ ^17^ ^,^ ^21^ ensuring the positive aspects of maintaining the practice of exercise during hospitalization, considering the other therapeutic benefits.

To date, the appropriate frequency, intensity, time, and type of activity for this population cannot be fully defined due to the variability of the protocols tested.^6^ ^,^ ^7^ ^,^ ^9^ ^,^ ^15^ ^,^ ^16^ ^,^ ^17^ ^,^ ^18^ ^,^ ^21^ ^,^ ^22^ While some studies started their interventions with a warm-up (cycling, walking, and stretching),^6^ ^,^ ^15^ ^,^ ^16^ most of them used strength training and aerobic training.^7^ ^,^ ^15^ ^,^ ^16^ ^,^ ^17^ ^,^ ^18^ ^,^ ^20^ ^,^ ^21^ Only the research by Speyer et al. (2010)^9^ used adapted activities, including games, dancing, interactive video games, weight training, among others. However, despite not stratifying their protocols in separated strength and aerobic exercises, the authors also used these characteristics indirectly. This procedure is in line with international recommendations, which advocate the daily practice of strength exercises and aerobic activities in children and adolescents.^37^

The present review had some limitations, including the different types of cancer (solid and hematologic) assessed in the studies, the different clinical severities, and the small sample size of each research. Furthermore, this work only included studies in English, Portuguese, and Spanish.

In conclusion, the findings of this review suggest that exercise improves muscle strength, physical fitness, and functional capacity in the short- and medium-term during hospitalization in children and adolescents with cancer. In addition, this practice proved to be safe, as long as the clinical aspects related to the disease are considered. We expect that professionals involved in cancer treatment try to implement exercise programs during hospital care, grounding their protocols at least on strength and aerobic training.
